# Effect of Xiongmatang Extract on Behavioral and TRPV1-CGRP/CGRP-R Pathway in Rats With Migraine

**DOI:** 10.3389/fphar.2022.835187

**Published:** 2022-03-08

**Authors:** Dingding Liu, Lulu Chang, Jingru Wang, Qiang Zhu, Ning Jiang, Mudassar Azhar, Ayaz Ahmed, Guirong Zeng

**Affiliations:** ^1^ College of Pharmacy, Guizhou University of Traditional Chinese Medicine, Guiyang, China; ^2^ Institute of Medicinal Plant Development, Chinese Academy of Medical Sciences and Peking Union Medical College, Beijing, China; ^3^ Dr. Panjwani Center for Molecular Medicine and Drug Research International Center for Chemical and Biological Sciences University of Karachi, Karachi, Pakistan; ^4^ Hunan Key Laboratory of Pharmacodynamics and Safety Evaluation of New Drugs and Hunan Provincial Research Center for Safety Evaluation of Drugs, Changsha, China

**Keywords:** migraine, xiongmatang extract, TRPV1-CGRP/CGRP-R pathway, behavioral, pathological

## Abstract

Migraine is a complex neurovascular disease, which seriously affects the quality of life in patients. This study aimed to evaluate the effect of Xiongmatang (XMT) extract on rats with migraine induced by inflammatory soup and the underlying mechanisms. First, 1 week after dural catheterization, inflammatory soup was injected through a microsyringe to stimulate the dura of rats for 6 times (12 days), once every 2 days, 10 *μ*L each time, to establish a migraine model. According to pain threshold analysis, behavioral change detection, and pathological analysis, the effects of XMT extract on rats with migraine were evaluated. The positive, mRNA and protein expression of related factors were detected by immunohistochemistry, RT-QPCR, and Western blot analysis to elucidate the underlying mechanism. XMT extract improved the behavioral performance of rats, and improve the pathological changes in the trigeminal nerve in rats. Further experimental results show that XMT extract regulated the expression of migraine-related factors in the trigeminal nerve, manifested as transient receptor potential vanilloid 1 (TRPV1), calcitonin-gene-related peptide (CGRP), calcitonin receptor-like receptor (CRLR), and receptor activity-modifying protein 1 (RAMP1) positive expression, mRNA expression, and protein expression reduction. XMT extract can significantly improved the behavioral performance of rats with migraine, and its mechanism of action might involve regulating the activity of TRPV1-CGRP/CGRP-R pathway.

## Introduction

Migraine is a highly disabling neurological disease, which seriously affects the quality of life in patients, which in turn imposes a heavy economic burden on the society (Ashina et al., 2021). Migraine is considered as a major public health challenge, occurring in about 18% of women and 6% of men (Harmon, 2015; Ashina et al., 2021). It has been listed as the world’s second largest cause of disability (Sun et al., 2017). In addition, migraine attacks are often accompanied by nausea, vomiting, photophobia, and other symptoms leading to anxiety, depression, cognitive disorders, and other diseases (Edvinsson et al., 2019).

At present, many drugs have been approved by the FDA for the treatment of migraine. Studies have shown Ubrogepant is safe and well tolerated over the long term (Ailani et al., 2020), but can cause adverse effects including nausea, drowsiness and dry mouth (Lipton et al., 2019a; Dodick et al., 2019). Rimegepant is approved for use in oral disintegrating tablets for acute migraine attacks. Rimegepant is fast-acting and has good initial efficacy, but is associated with adverse effects such as nausea and urinary tract infection (Lipton et al., 2019b). Erenumab is the first monoclonal antibody against CGRP approved by the FDA. Erenumab is safe and effective over the long term, but has been associated with injection site reactions, constipation, muscle cramps and other adverse reactions (Ashina et al., 2019a). Meanwhile, they may not be a cost-effective option for patients with low-frequency episodic migraine. Therefore, it is essential to explore new strategies (options) to cope with the migraine attacks. In recent years, studies have displayed that traditional Chinese medicines demonstrated better preventive and therapeutic outcomes in migraine. Studies further indicated that migraine-related changes, such as CGRP, inflammation, nitric oxide (NO) increased, and spontaneous behavior problems reduced by *Uncariae Ramulus Cum Uncis* extraction and its active constituents (Cheong-Meng et al., 2021). Tou Feng Yu pill (TFY), consisting of *Angelicae dahuricae* (Baizhi), *Ligusticum chuanxiong* (Chuanxiong) and *Folium Camelliae sinensis* (green tea), significantly reduced CGRP, NO, and brain dopamine (DA) levels of plasma in migraine rats (Jia-chuan et al., 2011). This study indicates gastrodin remarkably reduced CGRP-ir (+) neuron, CGRP -mRNA and pERK1/2 expression level in cultured rat trigeminal ganglion, effective treatment for migraine (Luo et al., 2011). Furthermore, senkyunolide I in Chuanxiong can regulate the levels of monoamine neurotransmitters and their turnover rate, as well as reduce NO levels in the blood and brain, and treat migraine headaches (Yi-Han et al., 2011). And they also exhibit advantage of being safe and inexpensive (Liu, 2014). Therefore, to explore the potential of traditional Chinese medicine for migraine treatment has attracted much attention.

XMT is a classical ancient prescription, comprising two medicines: *L. Chuanxiong* and *G. Elata*. The main chemical components of XMT are ligustrazine, ferulic acid, senkyunolide, and gastrodin (Chang et al., 2021). It was found that ligustrazine, ferulic acid, and senkyunolide could effectively treat migraine by reducing the protein content and CGRP-mRNA expressionin serum and cerebral cortex (Sha et al., 2019). Ferulic acid significantly reduced the number of CGRP stained cells in peripheral nerve injury (Sheng Chi et al., 2013). Moreover, gastrodin derivative (Gas-D) inhibited the peripheral release of CGRP after trigeminal nerve activation and NO clearance (Wang et al., 2016). Our previous studies showed that XMT effectively reduced the CGRP content in the plasma and brain tissue of rats with migraine (Liu et al., 2014; Liu et al., 2020a). This formulation also decreased the CGRP immunopositive neurons along with the activity of CGRP-CRLR/RAMP1 signaling pathway in the trigeminal nerve and spinal cord complex (TCC) (Liu et al., 2017). Recent research found, in the trigeminal ganglion, TRP channels co-localize with CGRP (Shibata and Tang, 2021), and the number of CGRP and TRPV1 immune reaction cells increase in the trigeminal ganglion of migraine rats (Chung-Chih et al., 2019). Our recent research found that XMT extract has also displayed a decline in the expression of TRPV1 on the trigeminal ganglion neurons of migraine rats (Liu et al., 2020b). Therefore, this experiment established migraine model rats with inflammation soup to explore whether the XMT extract works by regulating the TRPV1-CGRP/CGRP-R pathway.

## Materials and Methods

### Inflammation Soup and XMT Extract

Inflammation soup is a compound preparation containing 1 mM histamine, 1 mM bradykinin, 1 mM serotonin, 0.1 mM prostaglandin E2, and a solvent, phosphate-buffered saline (PBS) of pH 7.

**FIGURE 1:**
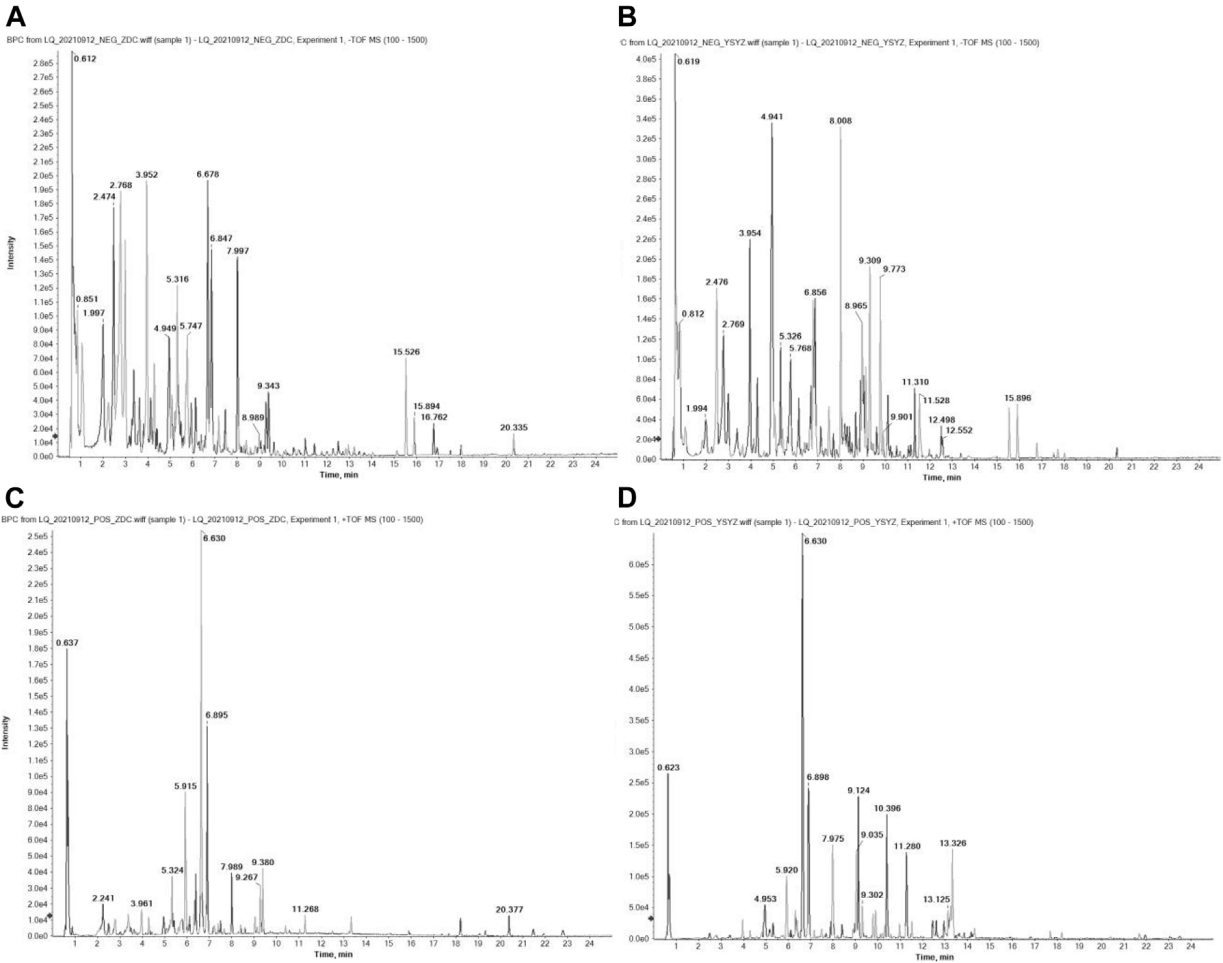
BPC diagram of XMT extract UHPLC-QTOF-MS detection in positive ion mode. **(A)** XMT nBuOH extract in negative ion mode; **(B)** XMT EtOAc extract in negative ion mode; **(C)** XMT nBuOH extract in positive ion mode; **(D)** XMT EtOAc extract in positive ion mode. The detection conditions are bombardment energy: 40 eV, collision energy difference: 20 V, 15 secondary spectrograms every 50 ms. ESI ion source parameters are set as follows: atomization pressure (GS1): 55 Psi, auxiliary pressure: 55 Psi, air curtain pressure: 35 Psi, temperature: 550°C, spray voltage: 5500 V (positive ion mode) or −4000 V (negative ion mode).

*L. Chuanxiong* and *G. Elata* were cut into small pieces and decoction was performed twice with eight times the amount of water successively for 45 min. After the decoction, the filtrate was extracted with petroleum ether, ethyl acetate, and *n*-butanol in a ratio of 1:1, and dried. The final amount of the extracts obtained were as follows: 95.9 g (3.8%) of petroleum ether extract, 93.5 g (3.7%) of ethyl acetate extract, and 38.1 g (1.5%) of *n*-butanol extract. The chemical components of XMT *n*BuOH and XMT EtOAc extracts were identified by UHPLC-QTOF-MS method (Figure 1).

### Animals

One hundred twenty SPF-grade SD male rats weighing 230–250 g were purchased from Hunan SJA Laboratory Animal Co., Ltd. The animals were raised in a SPF environment of Key Laboratory of Hunan Provincial Research Center for Safety Evaluation of Drugs. The ambient temperature was 18–26°C, and the humidity was 40–70%. All the rats were housed with 12 h of light or dark cycle and granted free access to standard food and tap water throughout the experiments. All the procedures were operated according to the Guide for the Care and Use of Laboratory Animals (Eighth Edition) issued by The National Academics (Washington D.C.) and Animal Ethics Committee of Hunan Provincial Research Center for Safety Evaluation of Drugs.

### Animal Modeling and Treatment

**FIGURE 2:**
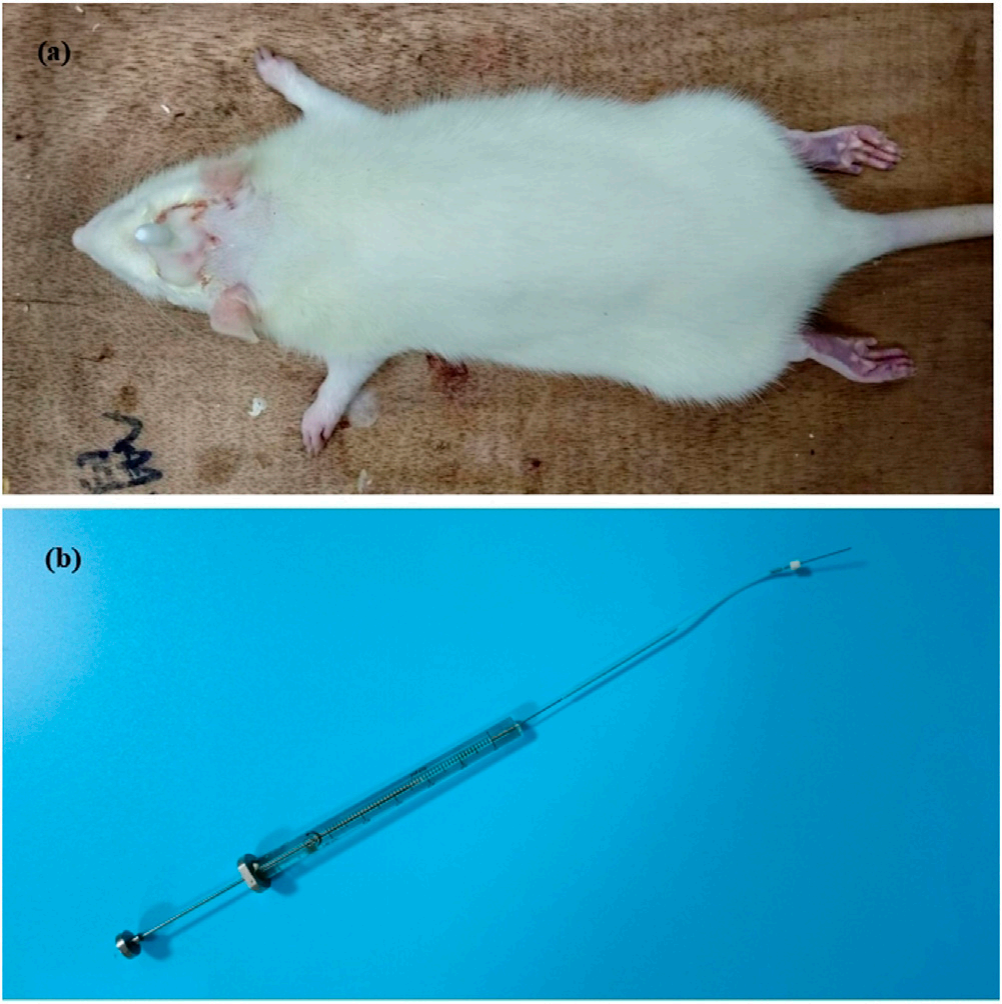
Dural catheterization in rats: **(A)** Catheterization result chart, **(B)** microsyringe.

**FIGURE 3:**
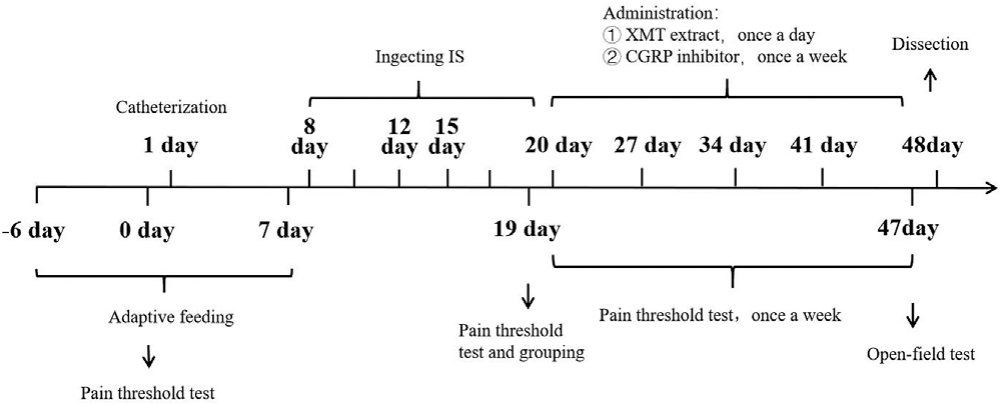
Experimental design.

First day, in SD rats, dura catheterization surgery was performed (Michael and Sumittra, 2007; Liu et al., 2021). Rats were anesthetized by inhalation of isoflurane. Then the hair on the top of the head was removed and fixed on the rat brain stereoscope, and the skin on the top of the head was fully disinfected with iodophor. The blade is then used to cut lengthwise through the skin on the top of the head, cutting through the subcutaneous fascia to fully expose the skull. Polish carefully with small tweezers to make it uneven and easy to fix the casing. Take a point 2 mm backward at the anterior fontanelle point and 2 mm to the right of the middle suture, and the diameter of the hole is 1 mm. With this hole as a reference, drill two identical holes (holes 2 and 3) behind the drilling hole, taking care not to drill the dura mater. Put the casing into hole 1, screw the screws into holes 2 and 3 respectively, and fix the casing with dental cement. Pay attention to dry the dental cement with a hair dryer in time (Figure 2). Later to 1 week (8 days), 90 rats with better living conditions were selected for micro-injecting inflammatory soup (10 *µ*L) through a microsyringe to stimulate the dura of rats for six times (12 days), once every 2 days, to establish a migraine model (Olivia et al., 2021), and another 10 rats with better living conditions were injected with saline in the same way. Then, 90 migraine rats were randomly divided into nine groups (n = 10/group): model (10 ml kg^−1^), CGRP inhibitor (rat CGRP-(8–37) (Bailey et al., 2012): MCE, Cat. No.: HY-P0209-85712; positive control, 10 μg kg^−1^), flunarizine (0.9 mg kg^−1^), XMT n-BuOH low dose (XMT *n*-BuOH—L, others are the same. 16.9 mg kg^−1^), XMT n-BuOH high dose (XMT *n*-BuOH—H, others are the same. 152.1 mg kg^−1^), XMT EtOAc—L (41.6 mg kg^−1^), XMT EtOAc—H (374.4 mg kg^−1^), XMT *n*-BuOH + EtOAc—L (58.5 mg kg^−1^), and XMT *n*-BuOH + EtOAc—H (526.5 mg kg^−1^). Another 10 rats were used as the control group. The control and model groups were administered pure water intragastrically (10 ml kg^−1^, i. s.). The CGRP inhibitor rats were anesthetized by isoflurane inhalation and fixed with a rat immobilizer, followed by tail vein injection of CGRP inhibitor (5 ml kg^−1^, i. v.), once a week, five times in total. In the meantime (20–47 days), the flunarizine, XMT *n*-BuOH—L, XMT *n*-BuOH—H, XMT EtOAc—L, XMT EtOAc—H, XMT *n*-BuOH + EtOAc—L, and XMT *n*-BuOH + EtOAc—H groups were given equal volume of the corresponding drugs by gavaging, once a day, for 4 weeks (28 days). The 48th day, the rats were sacrificed by isoflurane anesthesia, exsanguinated from the abdominal aorta, and the trigeminal nerve was dissected from the cheeks on both sides of the face. The left side was preserved in formalin solution for pathological examination and immunohistochemical staining. The right side was divided into two parts, which were frozen in liquid nitrogen, and used for RT-QPCR and Western blot detection. The experimental design is shown in Figure 3.

### Pain Threshold Test

The facial mechanical pain threshold of rats was measured by pain threshold instrumentat normal time and at 0, 1, 2, 3 and 4 weeks (19, 26, 33, 40s, and 47 days) of administration, respectively. The method is as follows: keep the rat in a quiet environment, gently hold the rat’s neck with one hand, hold its buttocks with the other hand, and ensure that its head can move freely. Then, the periorbital region was stimulated with an electronic von Frey monofilament (ElectrovonFrey 2,391, IITC Inc., Woodland Hills, CA, United States). The bending force of the von Frey apparatus was ranged from 0 to 800 g, and the sensitivity of this apparatus is 0.1 g. The stimuli were gradually increased to investigate the response thresholds. Positive responses were defined as quick retraction of the head upon stimulation or scratching of the face with the forepaw. The pressure thresholds were recorded automatically, and at each site, the test was repeated three times with an interval of at least 1 min (Feng et al., 2019).

### Open Field Test

After the last administration, all animals were subjected to the open field test only once. To start each session, a rat was placed in a particular corner of the open field and allowed to explore for 5 min. The open field apparatus consisted of a square arena (40 × 40 cm) with walls 40 cm high that was made of black polyvinyl chloride plastic board (Shanghai Jiliang Software Technology Co., Ltd., Shanghai, China). Their movement trajectories during 5 min were recorded by a video camera placed 145 cm above the arena and analyzed using JLBehv animal behavior analysis system (Shanghai Jiliang Software Technology Co., Ltd., Shanghai, China), and collected activity-related metrics, including the total distance, middle area activity distance, middle area activity time, and number activities. The apparatus was cleaned with 70% ethanol before the test of each animal (Hiroshi et al., 2017).

### Hematoxylin-Eosin Staining

The trigeminal nerve of rats fixed with 4% paraformaldehyde were trimmed, dehydrated, embedded, sliced, stained, and sealed following the pathological examination methods to prepare qualified microscopic specimens. The specimens were placed under a microscope for detailed observation of each tissue section, and films were taken at different multiples.

### Immunohistochemical Staining

The positive expression of TRPV1, CGRP, CRLR, and RAMP1 in the trigeminal nerve of rats was detected using the routine immunohistochemistry SABC method. Briefly, the tissue section was de-waxed, and incubated with 3% H_2_O_2_ solution (5–10 min) to quench the peroxidase activity. The tissues were processed for the antigen retrieval procedure. The primary antibody (mouse TRPV1 antibody: Proteintech, Cat. No.: 66983-1-Ig; rabbit CGRP antibody: Abclonal, Cat. No.: A5542; rabbit CRLR antibody: Abcam, Cat. No.: ab84467; rabbit RAMP1 antibody: Proteintech, Cat. No.: 10327-1-AP) was applied at 4°C for overnight followed by washing. These tissues were incubated with the secondary anitibody (anti-rabbit IgG) for 20 min at room temperature. SABC-Peroxidase was applied followed by the addition of chromogenic agents. The results were photographed using a Motic B5 microphotography system, and three pictures of each film were taken under 100× and 400× magnifications. The area of immunopositive staining was analyzed using an ImageJ medical analysis system.

### RT-QPCR Analysis

First, the total RNA was extracted from the trigeminal nerve of rats following the instructions of Trizol reagent manufacturer. Then, the total mRNA was used as the template for the reverse transcription of cDNA, and an RT-QPCR experiment was conducted. According to the sequences of rat TRPV1, CGRP, CRLR, RAMP1, and cDNA, Primer 5.0 software was used to complete the design of specific primers for TRPV1, CGRP, CRLR, and RAMP1 fragments (Shanghai Synthetical Primer, *β*-actin gene was used as the internal reference gene), and the primer sequence was as follows: *β*-actin: F ACA​TCC​GTA​AAG​ACC​TCT​ATG​CC, R TAC​TCC​TGC​TTG​CTG​ATC​CAC (Product length = 223); TRPV1: F CCC​GGA​AGA​CAG​ATA​GCC​TGA, R TTC​AAT​GGC​AAT​GTG​TAA​TGC​TG (product length = 92); CGRP: F TTC​CTG​GTT​GTC​AGC​ATC​TT, R GCTCCCTGGCTTTCATCT (product length = 166); CRLR: F CAA​TCA​TCC​ACC​TCA​CGG, R CCA​GAA​GTA​GTT​ACA​GCC​CAT​C (product length = 116); RAMP1: F GCT​GCT​GGC​TCA​CCA​TCT​C, R GCG​TCT​TCC​CAA​TAG​TCT​CCA (product length = 119). RT-QPCR and electrophoresis analyses of amplified DNA products were performed. The amplification conditions were as follows: 1 cycle for 10 min at 95°C, 15 s at 95°C, 30 s at 60°C, 40 cycles. Three multiple holes were made for each target gene and internal reference, and 2 (−△△CT) was used to represent the relative expression levels of TRPV1, CGRP, CRLR, RAMP1, and cDNA genes.

### Western Blot Analysis

First of all, before the formal experiment, a small amount of antibody should be taken for W-B detection to effectively confirm whether the antibody specifically binds to the target protein according to the molecular weight. Then, trigeminal nerve of rats (0.015 g) was cut and washed with precooled PBS. 200 *μ*L of RIPA lysate was added for 10 min for ice lysis, and the mixture was centrifuged at 12,000 rpm for 15 min at 4°C. The supernatant was taken, and the protein concentration was quantitatively measured. Then, the following steps were carried out: spot sampling, the denatured protein was subjected to sodium dodecyl sulfate-polyacrylamide (SDS-PAGE) gel electrophoresis (concentrating gel electrophoresis at 80 V, separating gel electrophoresis at 120 V, 150 min), membrane transfer (at a constant current of 300 mA, TRPV1 was 120 min; CGRP and RAMP1 were 30 min; CRLR was 80 min; and *β*-actin was 60 min), sealed for 90 min, incubated with primary antibody (maintained overnight at 4°C and left at room temperature for 30 min the next day; mouse TRPV1 antibody (Proteintech, Cat. No.: 66983-1-Ig) was diluted at 1:1,000; rabbit CGRP antibody (Abclonal, Cat. No.: A5542) was diluted at 1:500; rabbit CRLR antibody (Abcam, Cat. No.: ab84467) was diluted at 1 *μ*g/ml; rabbit RAMP1 antibody (Proteintech, Cat. No.: 10327-1-AP) was diluted at 1:500; and mouse *β*-actin antibody (Proteintech, Cat. No.: 66009-1-Ig) was diluted at 1:5,000), and incubated with secondary antibody (90 min, the dilution ratio of HRP goat anti-mouse IgG was 1:5,000; and the dilution ratio of HRP goat anti-rabbit IgG was 1:6,000). Finally, the film was incubated with an enhanced chemiluminescence (ECL) solution for 1 min, and the film was exposed with an X film for 20 min in a dark box. The developed film was rinsed. The protein expressions of TRPV1, CGRP, CRLR, and RAMP1 in the trigeminal nerve of rats were detected, 3 times per sample. Relative protein expression intensity = target protein expression intensity/*β*-actin protein expression intensity.

### Statistical Analysis

All data were analyzed using SPSS version 26.0. Data are presented as mean ± standard error (*SE*). Multiple comparisons were used with one-way or two-way ANOVA. Prior to the ANOVA test, results were first assessed for the normality of the residuals and homogeneity of variance. Differences were considered to be significant at *p* < 0.05.

## Results

### Pain Threshold in Rats

**FIGURE 4:**
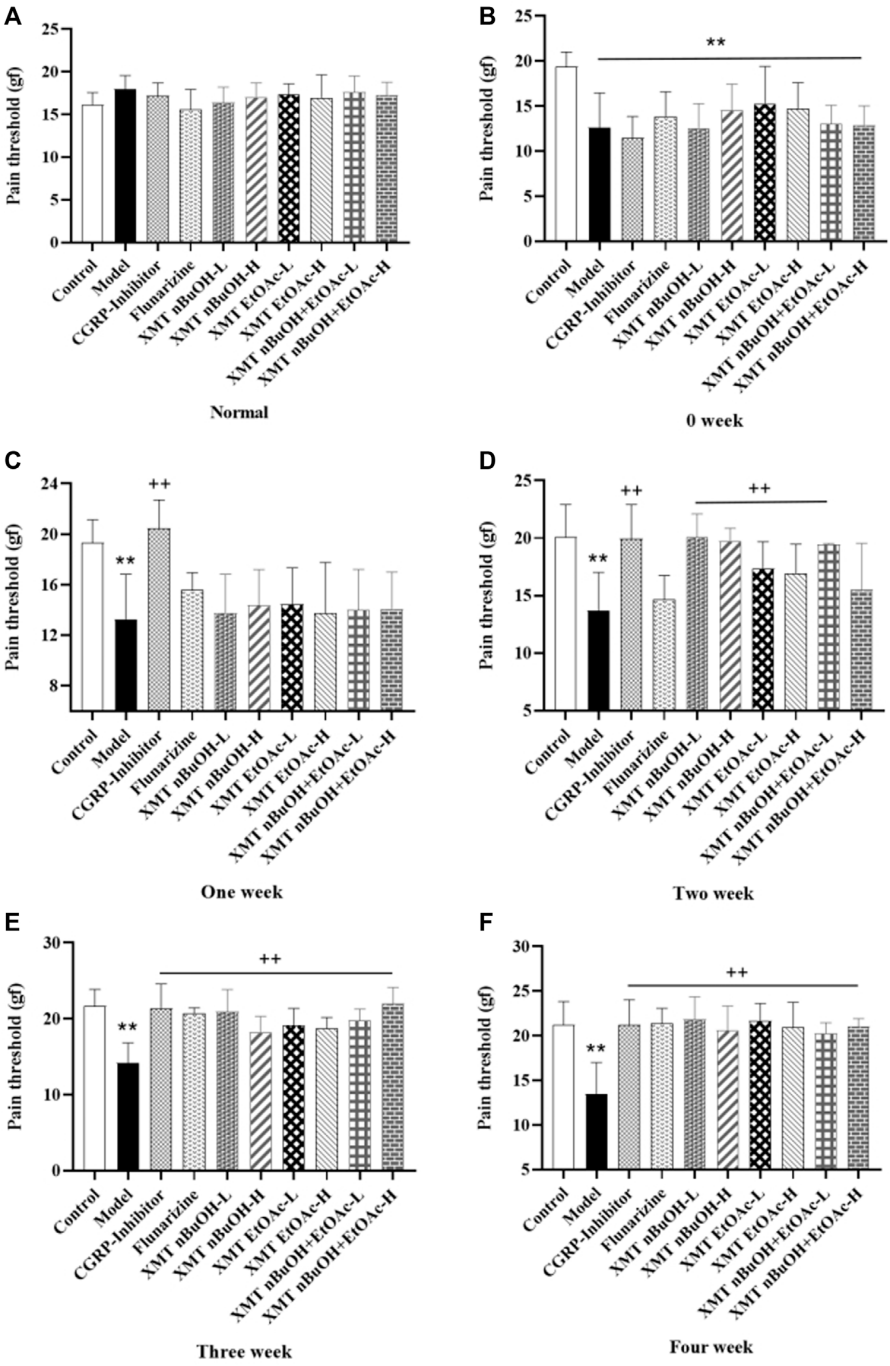
Results of pain threshold test in rats. **(A)** Normal, **(B)** 0 weeks, **(C)** One week, **(D)** Two week, **(E)** Three week, **(F)** Four week. All values are expressed as mean ± S.D. Different symbols indicate significant differences (compared with the control group, ^**^ *p* < 0.01; compared with the model group, ^+^ *p* < 0.05, ^++^ *p* < 0.01).

Before the treatment, the pain threshold of rats in the model, CGRP inhibitors, flunarizine, XMT *n*-BuOH—L, XMT *n*-BuOH—H, XMT EtOAc—L, XMT EtOAc—H, XMT *n*-BuOH + EtOAc—L, and XMT *n*-BuOH + EtOAc—H groups was significantly lower than that of the control group (*p* < 0.01). During the 4 weeks of treatment, the pain threshold of the treatment group showed an upward trend. After the last administration, the pain threshold of rats in the model group was significantly lower than that of the control group (*p* < 0.01). Compared with the model group, the pain threshold of rats in CGRP inhibitors, flunarizine, XMT *n*-BuOH—L, XMT *n*-BuOH—H, XMT EtOAc—L, XMT EtOAc—H, XMT *n*-BuOH + EtOAc—L, and XMT *n*-BuOH + EtOAc—H groups significantly increased (*p* < 0.01) (Figure 4, Supplementary Table S1).

### Open Field Activity in Rats

**FIGURE 5:**
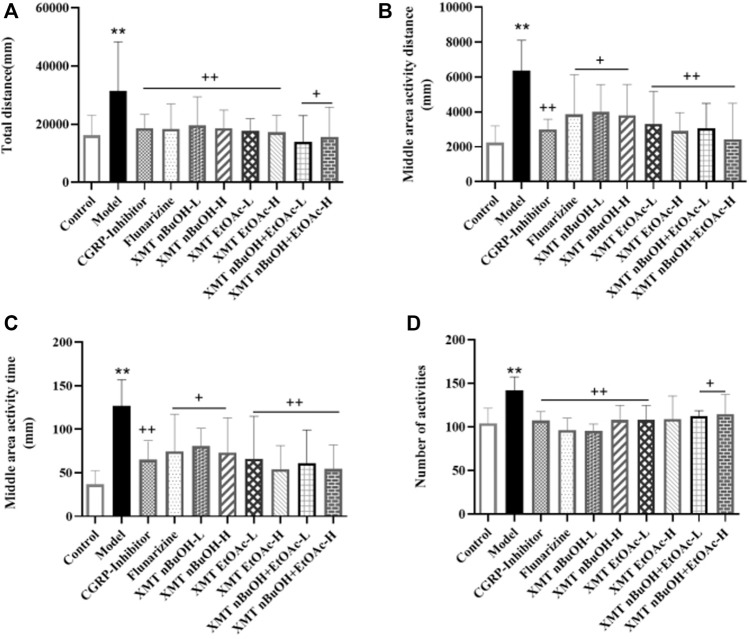
Results of open field test: **(A)** total distance, **(B)** middle activity distance, **(C)** middle area activity time, **(D)** number activities. All values are expressed as mean ± S.D. Different symbols indicate significant differences (compared with the control group, ^**^ *p* < 0.01; compared with the model group, ^+^ *p* < 0.05, ^++^ *p* < 0.01).

In the open field test, the total distance, middle area activity distance and time, and number activities of model rats significantly increased compared with the control group (*p* < 0.01). Compared with the model group, the total distance, middle area activity distance and time, and number activities of rats in CGRP inhibitors, flunarizine, XMT *n*-BuOH—L, XMT *n*-BuOH—H, XMT EtOAc—L, XMT EtOAc—H, XMT *n*-BuOH + EtOAc—L, and XMT *n*-BuOH + EtOAc—H groups significantly decreased (*p* < 0.01, *p* < 0.05) (Figure 5).

### Pathological Changes in the Trigeminal Nerve in Rats

**FIGURE 6:**
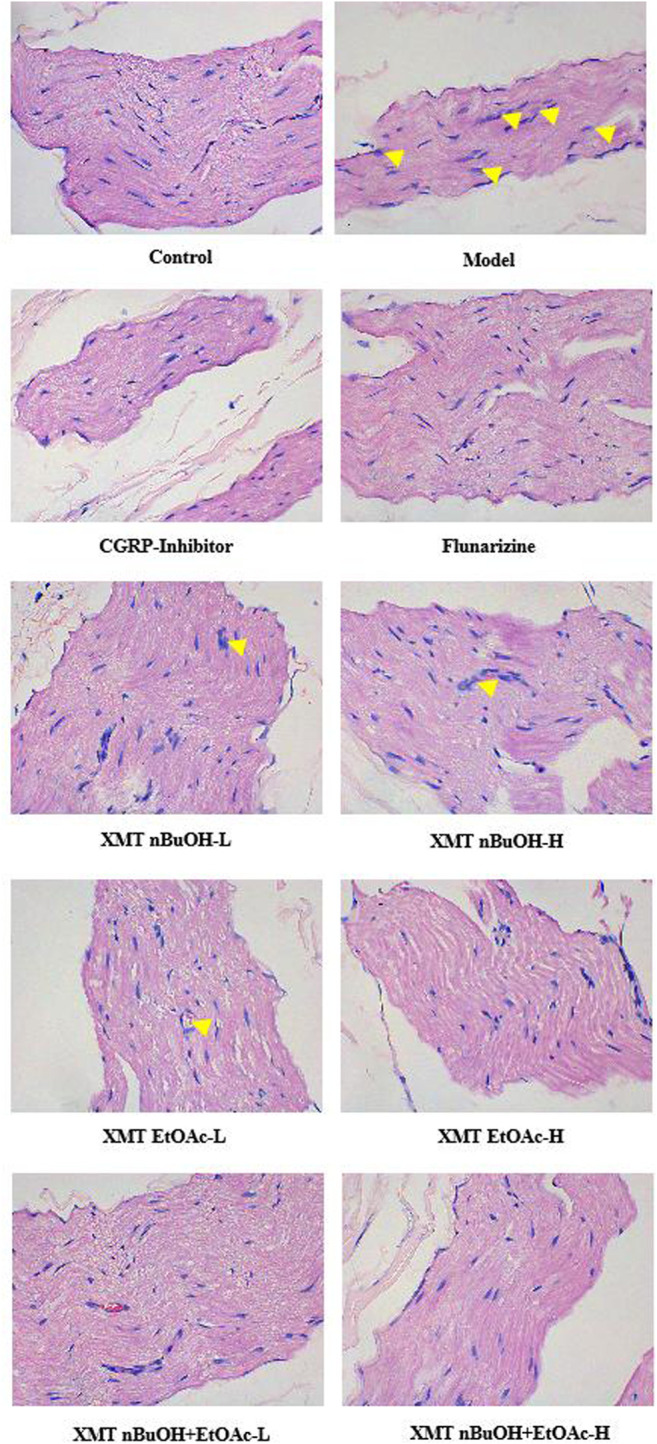
Results of HE staining (detecting the pathological structural changes of the trigeminal nerve). The pictures were taken under 400× magnification. The yellow arrows represent nuclear disintegration.

In the pathological examination, the trigeminal nerve fibers of control rats were dense, and the nerve cells were arranged in an orderly manner. Nevertheless, in the model group, the structure of the trigeminal nerve was disordered, and the nucleus was disintegrated. The pathological changes of each administration group improved. Among them, the nerve cells in the CGRP inhibitor group, flunarizine group, XMT EtOAc—H, XMT n-BuOH + EtOAc—L, and XMT n-BuOH + EtOAc—H groups were neatly arranged, the nerve fibers were dense, and no nucleus was found crack. The cells in the XMT n-BuOH—L, XMT n-BuOH—H and XMT EtOAc—L groups were neatly arranged, and some nuclei were still disintegrated (Figure 6).

### Immunohistochemical Analysis

**FIGURE 7:**
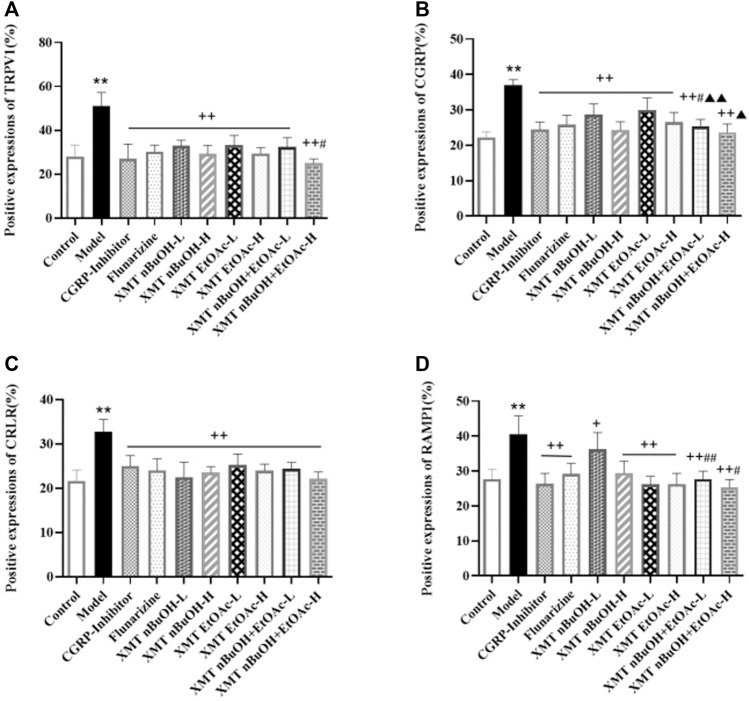
Results of positive expression of TRPV1, CGRP, CRLR, and RAMP1 in the trigeminal nerve of rats. **(A)** Positive expression of TRPV1, **(B)** Positive expression of CGRP, **(C)** Positive expression of CRLR, **(D)** Positive expression of RAMP1. All the values are expressed as mean ± S.D. Differentsymbols indicate significant differences (compared with the control group, ^**^ *p* < 0.01; compared with the model group, ^+^ *p* < 0.05, ^++^ *p* < 0.01; compared with the same dose of XMT *n*-BuOH, ^#^ *p* < 0.05, ^##^ *p* < 0.01; compared with the same dose of XMT EtOAc, ^▲^ *p* < 0.05, ^▲▲^ *p* < 0.01).

Compared with the control group, the positive expressions of TRPV1, CGRP, CRLR, RAMP1 in the trigeminal nerve of model group significantly increased (*p* < 0.01). Compared with the model group, the positive expressions of rats’ TRPV1, CGRP, CRLR, and RAMP1 in CGRP inhibitor, flunarizine, XMT *n*-BuOH—H, XMT EtOAc—L, XMT EtOAc—H, XMT *n*-BuOH + EtOAc—L, XMT *n*-BuOH + EtOAc—H groups and the positive expressions of TRPV1, CGRP, and CRLR in XMT *n*-BuOH—L group significantly decreased (*p* < 0.01). The positive expressions of RAMP1 in the trigeminal nerve of rats in XMT *n*-BuOH—L group decreased (*p* < 0.05). Moreover, the positive expression of CGRP in XMT *n*-BuOH + EtOAc—L group was significantly lower than that in XMT *n*-BuOH—L and XMT EtOAc—L group (*p* < 0.05, *p* < 0.01), and the positive expression of RAMP1 was significantly lower than that of XMT *n*-BuOH—L group (*p* < 0.01). The positive expression of TRPV1 and RAMP1 in XMT *n*-BuOH + EtOAc—H group was significantly lower than that of XMT *n*-BuOH—H group (*p* < 0.05), and the positive expression of CGRP was significantly lower than that of XMT EtOAc—H (*p* < 0.05) (Figure 7, Supplementary Figures S1–S4).

### RT-QPCR Analysis

**FIGURE 8:**
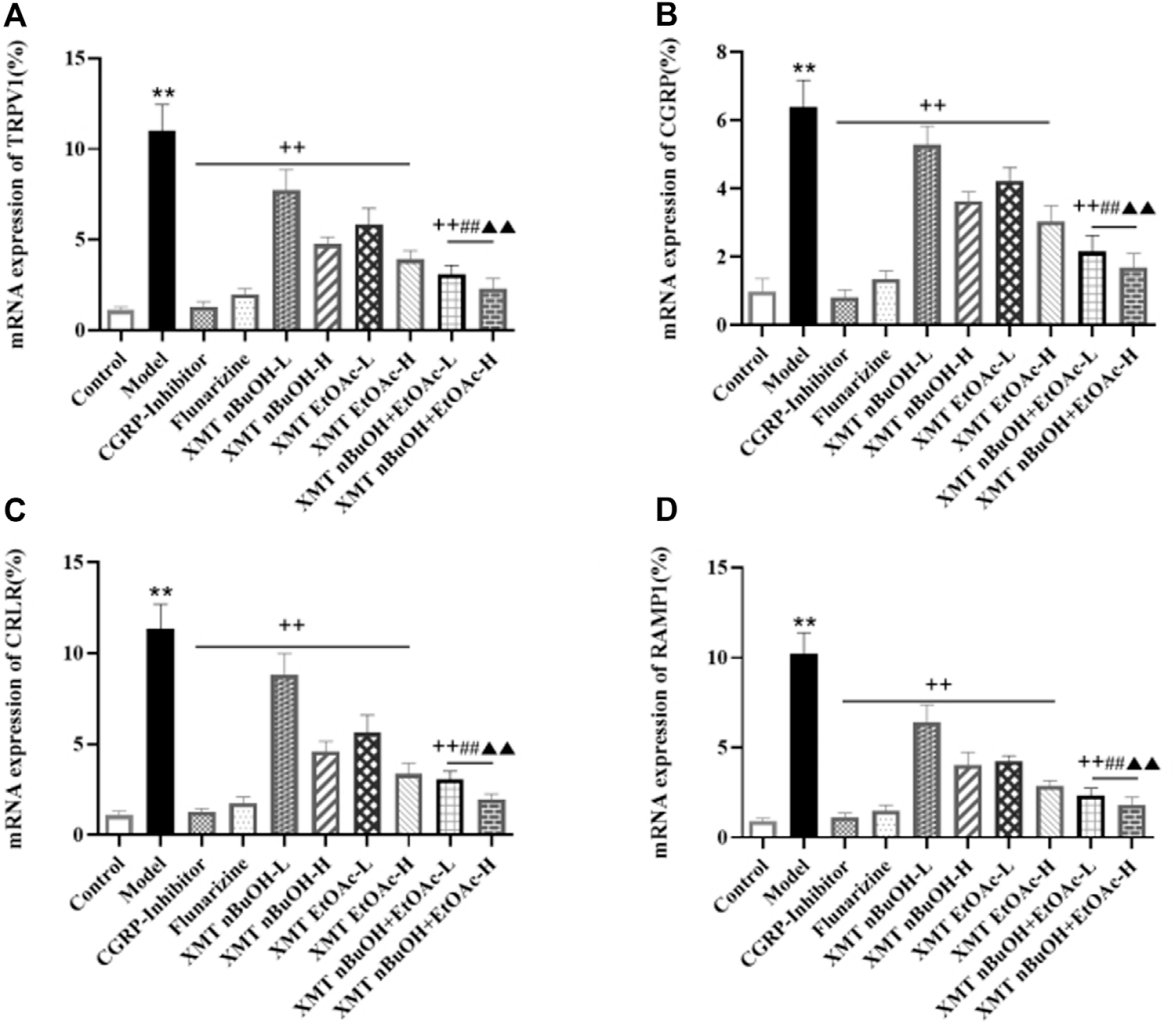
Results of mRNA expression of TRPV1, CGRP, CRLR, and RAMP1 in the trigeminal nerve of rats. **(A)** mRNA expression of TRPV1, **(B)** mRNA expression of CGRP, **(C)** mRNA expression of CRLR, **(D)** mRNA expression of RAMP1. All the values are expressed as mean ± S.D. Differentsymbols indicate significant differences (comparedwith the control group, ^**^ *p* < 0.01; compared with the model group, ^++^ *p* < 0.01; compared with the same dose of XMT *n*-BuOH, ^##^ *p* < 0.01; compared with the same dose of XMT EtOAc, ^▲▲^ *p* < 0.01).

In RT-QPCR testing, mRNA expressions of TRPV1, CGRP, CRLR, and RAMP1 in model rats were higher than the control group (*p* < 0.01). The mRNA expressions of TRPV1, CGRP, CRLR, and RAMP1 for CGRP inhibitor, flunarizine, XMT *n*-BuOH—L, XMT *n*-BuOH—H, XMT EtOAc—L, XMT EtOAc—H, XMT *n*-BuOH + EtOAc—L, and XMT *n*-BuOH + EtOAc—H groups in the trigeminal nerve measurement significantly decreased (*p* < 0.01). Furthermore, the mRNA expression of TRPV1, CGRP, CRLR, and RAMP1 in XMT *n*-BuOH + EtOAc—L group was significantly lower than that in XMT *n*-BuOH—L and XMT EtOAc—L groups (*p* < 0.01). The mRNA expression of TRPV1, CGRP, CRLR, and RAMP1 in XMT *n*-BuOH + EtOAc—H group was significantly lower than that in the XMT *n*-BuOH—H and XMT EtOAc—H groups (*p* < 0.01) (Figure 8).

### Western Blot Analysis

**FIGURE 9:**
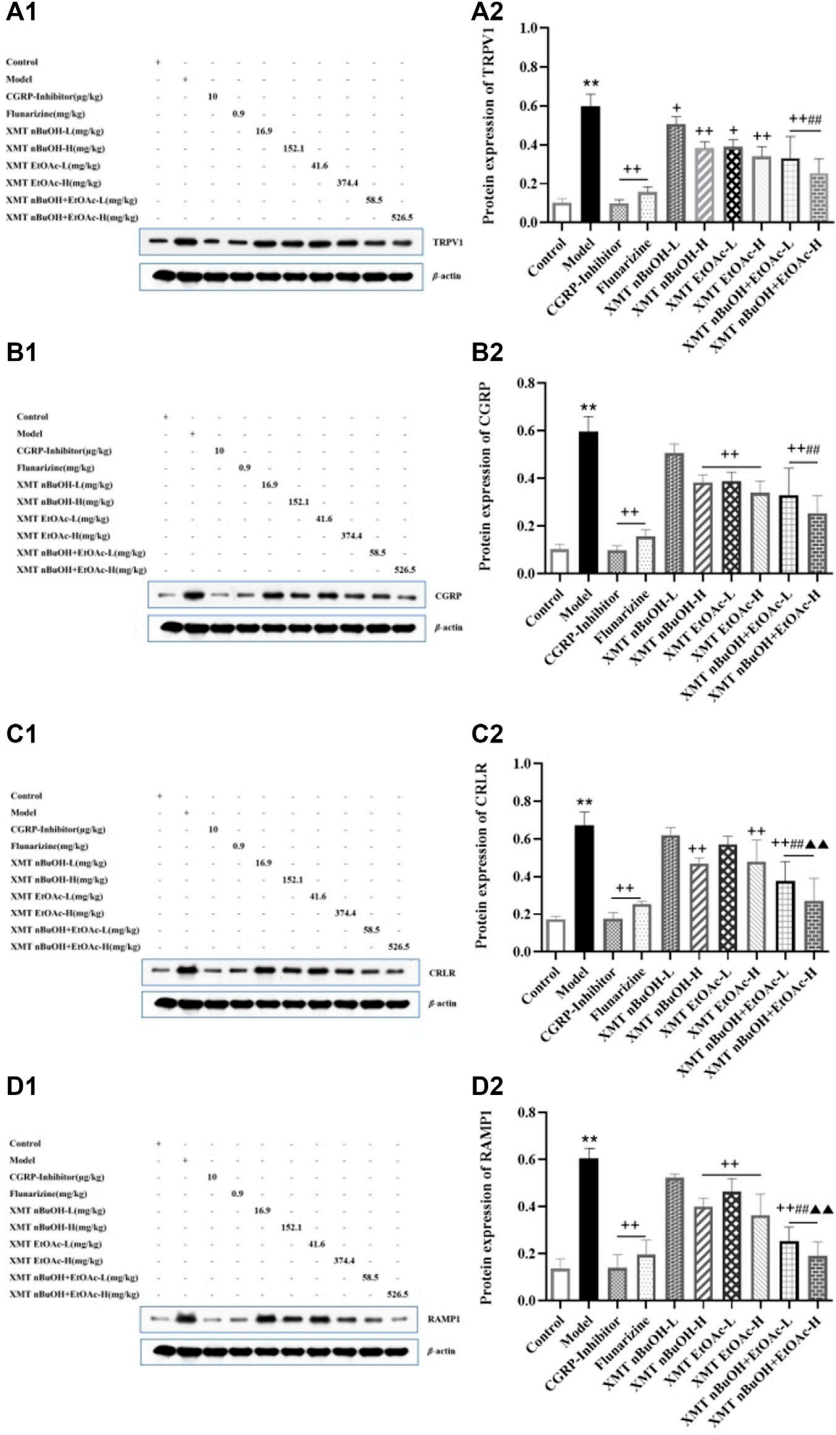
Xiongmatang extract protein expression of TRPV1, CGRP, CRLR, and RAMP1 in the trigeminal nerve of rats. **(A)** Protein expression of TRPV1, **(B)** Protein expression of CGRP, **(C)** Protein expression of CRLR, **(D)** Protein expression of RAMP1. All the values are expressed as mean ± S.D. Different symbols indicate signifi cant differences (compared with the control group, ^**^ *p* < 0.01; compared with the model group, ^+^ *p* < 0.05, ^++^ *p* < 0.01; compared with the same dose of XMT *n*-BuOH, ^##^ *p* < 0.01; compared with the same dose of XMT EtOAc, ^▲▲^ *p* < 0.01).

The protein expression of TRPV1, CGRP, CRLR, and RAMP1 in rats’ trigeminal nerve of model group significantly increased compared with the control group (*p* < 0.01). Compared with the model group, the protein expression of TRPV1, CGRP, CRLR, and RAMP1 in CGRP inhibitor, flunarizine, XMT *n*-BuOH—H, XMT EtOAc—H, XMT *n*-BuOH + EtOAc—L, XMT *n*-BuOH + EtOAc—H groups and positive expression of CGRP and RAMP1 in XMT EtOAc—L groups was lower (*p* < 0.01). The protein expression of TRPV1 in rats’ trigeminal nerves in XMT *n*-BuOH—L and XMT EtOAc—L group decreased (*p* < 0.05). Besides, the protein expression of TRPV1 and CGRP in XMT *n*-BuOH + EtOAc—L group was significantly lower than that in XMT *n*-BuOH—L, and the protein expression of CRLR and RAMP1 was significantly lower than that in XMT *n*-BuOH—L and XMT EtOAc—L groups (*p* < 0.01). The protein expression of TRPV1 and CGRP in XMT *n*-BuOH + EtOAc—H group was significantly lower than that in XMT *n*-BuOH—H, and the protein expression of CRLR and RAMP1 was significantly lower than that in XMT *n*-BuOH—H and XMT EtOAc—H groups (*p* < 0.01) (Figure 9).

## Discussion

At present, the pathogenesis of migraine is still unclear, and the theory of the trigeminal neurovascular system is the modern accepted theory of the pathogenesis of migraine. It is believed to be the anatomical and physiological basis for nociceptive transmission and for migraine sensation (Noseda and Burstein, 2013; Ashina et al., 2019b). And the brain stem activates TRPV1 nociceptors around the trigeminal nerve endings around the cerebral blood vessels by receiving stimulation from the thalamus, hypothalamus and cortex, releasing CGRP, substance P (SP) and other vasoactive peptides, causing neurogenic inflammation of the dura and other trigeminal nerve distribution tissues (Melo-Carrillo et al., 2017; Shibata and Tang, 2021).

TRPV1 belongs to the transient receptor potential (TRP) family, also known as capsaicin receptor, which can be activated by chemical components such as capsaicin, pH < 6, temperature >42 and SP. This channel causes the influx of cations such as Ca^2+^ and the efflux of K^+^, which leads to the release of SP and CGRP, causing nociceptive pain perception. And over the past 30 years, both clinical and preclinical studies have documented the role of the neuropeptide CGRP, the most potent vasodilatory peptide known (Anne-Sophie, et al., 2020). In the same time, it is an inflammatory marker, involved in the occurrence and development of neurogenic inflammation (Satyanarayana et al., 2018; Edvinsson, 2019). Althoughthe mechanism and the genetic background that by promoting cranial neurogenic vasodilatation generate migraine pain remain a mystery, A series of agents known to provoke migraine attacks have been identified as activators of certain transient receptor potential (TRP) channels expressed by a subpopulation of peptidergic nociceptors. In particular, the subtypes ankyrin 1 (TRPA1) and vanilloid 1 (TRPV1) are activated by migraine provoking agents. Nitric oxide, umbellulone and acrolein gate TRPA1 and alcohol TRPV1. All stimuli by channel targeting generate CGRP release from perivascular terminals of cranial sensory neurons, thus producing the neurogenic effect blunted by CGRP-R antagonists and by anti CGRP mAbs (Pierangelo, et al., 2015). It is concluded that by inhibiting the release of CGRP in the TRPV1-CGRP/CGRP-R signaling pathway in the trigeminal neurovascular system of migraine, it can provide a new strategy for migraine treatment.

Based on the above studies, it is shown that stimulation of the trigeminal neurovascular system is considered to be the initial event of the physiological cascade that leads to migraine (Margaret and Constance, 2011). Moreover, dural inflammation is considered to be the cause of activation of trigeminal neurovascular system (Débora et al., 2016). Studies have shown that the injection of inflammation soup through the dura mater could induce hyperalgesia in rats, causing meningeal neurogenic inflammatory pain sensitivity migraine and significantly reducing the mechanical pain threshold in rats (Liu et al., 2021; Olivia et al., 2021). According to the results obtained in this study, the pain threshold of rats was significantly reduced after 2 weeks of modeling with inflammation soup. Another study showed that various types of sensory pain thresholds in the pain areas reduced during the migraine attack (Débora et al., 2016). The pain thresholdTRPV1 of frontal muscle and bilateral posterior temporal muscle was significantly reduced in migraine patients (Rogers et al., 2020). Pharmacological experiments showed that the mechanical pain threshold of rats with migraine was significantly reduced (Liu et al., 2021). The results of this study are consistent with these results reported in the literature. At the same time, studies have shown that migraines are associated with anxiety-like behavior (Sun et al., 2017). The movement distance of the peripheral area of rats with migraine and anxiety increased significantly (Ke-Zhu et al., 2015). The results of this study also showed that the total distance, middle area activity distance and time, and number of activities in the model group rats significantly increased. Furthermore, 4 weeks after administering the XMT extract, compared with the model group, the pain threshold of rats in each administration group was significantly increased; at the same time, the total distance, middle area activity distance and time, and number of activities significantly reduced. Therefore, it is suggested that the XMT extract has a certain therapeutic effect on migraine.

Besides, The canonical CGRP receptor consists of CRLR and RAMP1 together. The study found CGRP, CRLR and RAMP1 are widely expressed in trigeminal nerve vessels (Iyengar et al., 2019). Activation of the trigeminal neurovascular system with nitroglycerin can increase the mRNA expression levels of TRPV1 and CGRP in the trigeminal ganglion of rats, which are significantly reduced after auxin release peptide mediation (Fereshteh et al., 2018). Other studies have shown that the activation of TRPV1 receptor plays an important role in the occurrence and development of migraine and the formation of hyperalgesia, and injection of TRPV1 receptor antagonist into the lateral ventricle of rats with migraine can provide analgesic effect (Jannis et al., 2015). When the TRPV1/Ca^2+^ signaling pathway is opened, Ca^2+^ and other cations will enter the intracellular matrix from the extracellular matrix, thus increasing the release of CGRP and other factors (Suckow et al., 2012). In this study, the positive expression, mRNA expression, and protein expression of TRPV1, CGRP, CRLR, and RAMP1 in the trigeminal nerve of migraine model rats significantly increased. This indicates that the occurrence of migraine might be closely related to the expression of TRPV1-CGRP/CGRP-R pathway in trigeminal nerve.

In the results of this study, XMT *n*-BuOH and XMT EtOAc extract showed good effects on migraine. Among them, the positive expression, mRNA expression and protein expression of TRPV1, CGRP, CRLR, and RAMP1 in XMT *n*-BuOH-H, XMT EtOAc-H, XMT *n*-BuOH + EtOAc—L, and XMT *n*-BuOH + EtOAc—H groups of rats significantly decreased. The effect from good to bad is as follows: XMT *n*-BuOH and XMT EtOAc, XMT *n*-BuOH + EtOAc. These results indicate that the XMT extracts, especially the XMT *n*-BuOH + EtOAc, effectively reduced the activity of TRPV1-CGRP/CGRP-R pathway in the trigeminal nerve of migraine rats.

## Conclusion

XMT extract could significantly improve the macroscopic performance and behavioral performance of migraine rats, its mechanism of action involves regulating the TRPV1-CGRP/CGRP-R pathway.
