# Supplementary material for: Effect of Xiongmatang Extract on Behavioral and TRPV1-CGRP/CGRP-R Pathway in Rats With Migraine
Source: Front Pharmacol. 2022 Mar 8;13:835187. doi: 10.3389/fphar.2022.835187 (PMC8957915; doi:10.3389/fphar.2022.835187)
Supplement: Supplementary file 1 [file DataSheet1.docx]

**Supplementary Material**

**1 Supplementary tables**

**Tab. 1.** Results of pain threshold test in rats ($\overline{x}\pm s$)

| Group | Pain threshold (gf) | | | | | |
| --- | --- | --- | --- | --- | --- | --- |
|  | normal | 0 week | One week | Two week | Three week | Four week |
| A | 16.17±1.41 | 19.36±1.647 | 19.31±1.83 | 20.10±2.82 | 21.67±2.20 | 21.27±2.57 |
| B | 17.98±1.59 | 12.66±3.81^**^ | 13.28±3.54^**^ | 13.70±3.31^**^ | 14.23±2.60^**^ | 13.50±3.52^**^ |
| C | 17.20±1.50 | 11.47±2.37^**^ | 20.47±2.25 ^++^ | 19.96±2.96 ^++^ | 21.42±3.16 ^++^ | 21.26±2.77 ^++^ |
| D | 15.58±2.36 | 13.85±2.75^**^ | 15.57±1.38 | 14.67±2.07 | 20.77±0.67 ^++^ | 21.40±1.68 ^++^ |
| E | 16.43±1.77 | 12.58±2.70^**^ | 13.75±3.08 | 20.07±2.04 ^++^ | 20.96±2.87 ^++^ | 21.86±2.51 ^++^ |
| F | 17.05±1.64 | 14.53±2.91^**^ | 14.38±2.81 | 19.78±1.08 ^++^ | 18.24±2.07 ^++^ | 20.62±2.73 ^++^ |
| G | 17.35±1.25 | 15.30±4.10^**^ | 14.45±2.88 | 17.38±2.31 ^++^ | 19.12±2.24 ^++^ | 21.67±1.95 ^++^ |
| H | 16.97±2.69 | 14.72±2.89^**^ | 13.75±4.01 | 16.93±2.55 ^+^ | 18.75±1.43 ^++^ | 20.92±2.83 ^++^ |
| I | 17.63±1.88 | 13.03±2.06^**^ | 14.00±3.21 | 19.45±0.07 ^++^ | 19.77±1.51^++^ | 20.27±1.20 ^++^ |
| J | 17.30±1.48 | 12.93±2.10^**^ | 14.10±2.91 | 15.53±4.02 | 21.97±2.17 ^++^ | 21.03±0.90 ^++^ |

A, Control; B, Model; C, CGRP inhibitor (10 µg·kg^−1^); D, flunarizine (0.9 mg·kg^−1^); E, XMT *n*-BuOH – L (16.9 mg·kg^−1^); F, XMT *n*-BuOH – H (152.1 mg·kg^−1^); G, XMT EtOAc – L (41.6 mg·kg^−1^); H, XMT EtOAc – H (374.4 mg·kg^−1^); I, XMT *n*-BuOH + EtOAc – L (58.5 mg·kg^−1^) ; J, XMT *n*-BuOH + EtOAc – H (526.5 mg·kg^−1^). Different symbols indicate significant differences (compared with the control group, ^**^*P* < 0.01; compared with the model group, ^+^*P* < 0.05, ^++^*P* < 0.01).

**2 Supplementary figures**


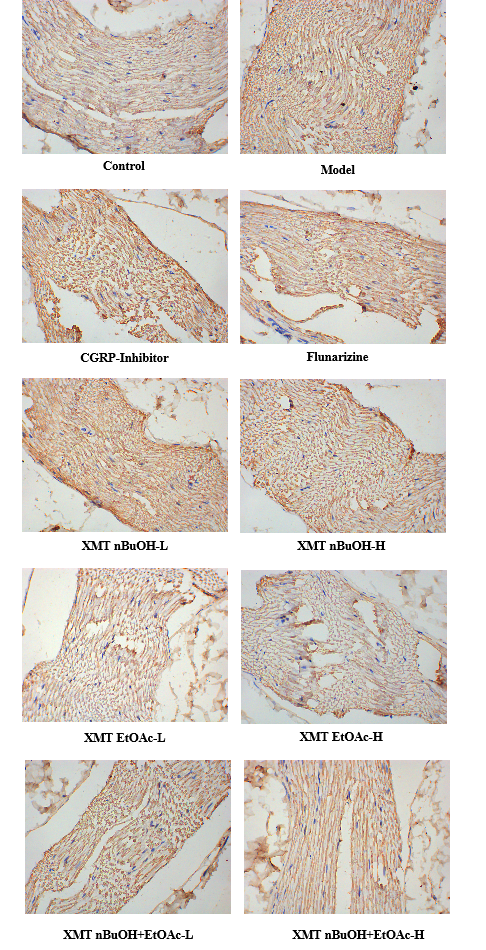


**Fig. 1.** TRPV1-like immunohistochemical staining. The pictures were taken under 400× magnification. The positive expression appears as yellow or tan brownish (dark brown) staining.


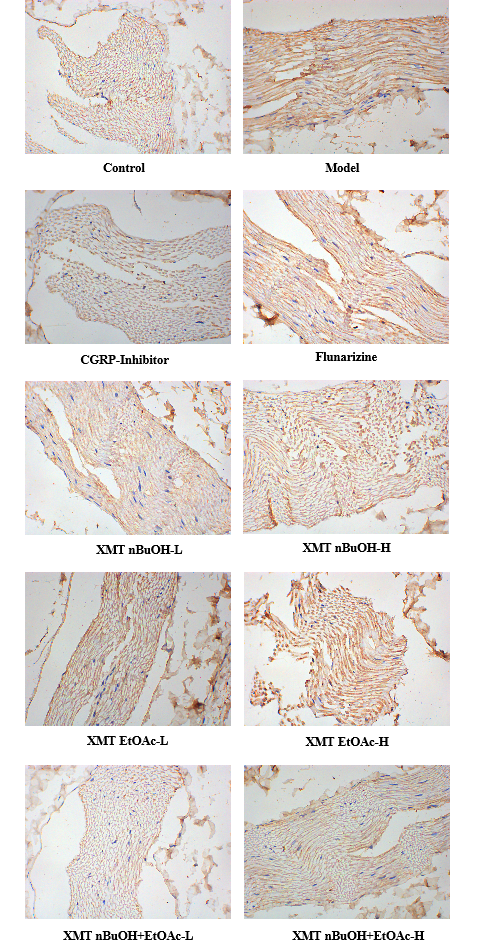


**Fig. 2.** CGRP-like immunohistochemical staining. The pictures were taken under 400× magnification. The positive expression appears as yellow or tan brownish (dark brown) staining.


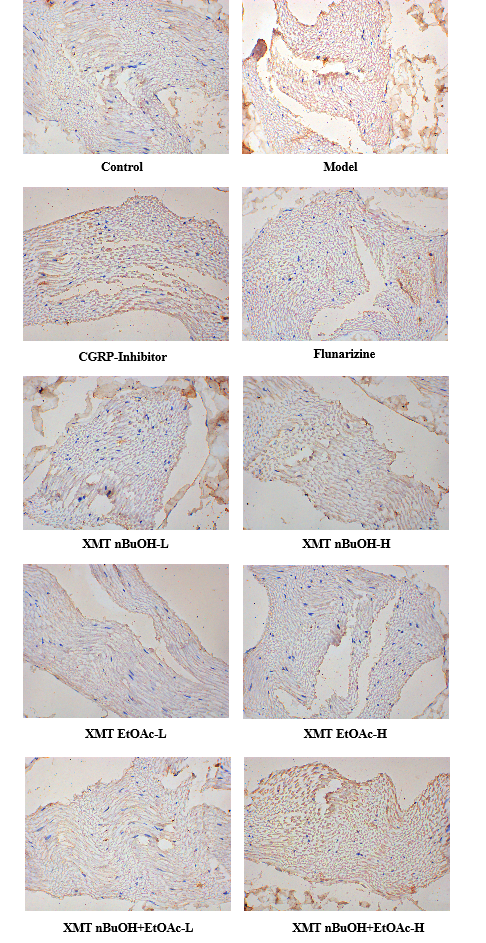


**Fig. 3.** CRLR-like immunohistochemical staining. The pictures were taken under 400× magnification. The positive expression appears as yellow or tan brownish (dark brown) staining.


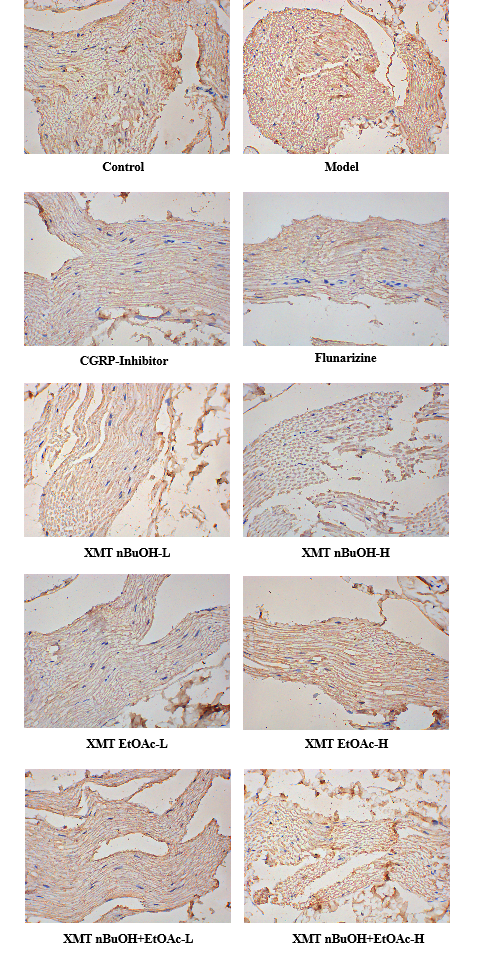


**Fig. 10.** RAMP1-like immunohistochemical staining. The pictures were taken under 400× magnification. The positive expression appears as yellow or tan brownish (dark brown) staining.
